# Preliminary Assessment of Sponge Biodiversity on Saba Bank, Netherlands Antilles

**DOI:** 10.1371/journal.pone.0009622

**Published:** 2010-05-21

**Authors:** Robert W. Thacker, M. Cristina Díaz, Nicole J. de Voogd, Rob W. M. van Soest, Christopher J. Freeman, Andrew S. Mobley, Jessica LaPietra, Kevin Cope, Sheila McKenna

**Affiliations:** 1 Department of Biology, University of Alabama at Birmingham, Birmingham, Alabama, United States of America; 2 Museo Marino de Margarita, Nueva Esparta, Venezuela; 3 Naturalis, National Museum of Natural History, Leiden, The Netherlands; 4 Zoological Museum of the University of Amsterdam, Amsterdam, The Netherlands; 5 Deep Search Foundation, Alameda, California, United States of America; 6 Ecology Centre and Commonwealth Facility for Applied Environmental Decision Analysis, University of Queensland, Brisbane, Australia; University of Zurich, Switzerland

## Abstract

**Background:**

Saba Bank Atoll, Netherlands Antilles, is one of the three largest atolls on Earth and provides habitat for an extensive coral reef community. To improve our knowledge of this vast marine resource, a survey of biodiversity at Saba Bank included a multi-disciplinary team that sampled fishes, mollusks, crustaceans, macroalgae, and sponges.

**Methodology/Principal Findings:**

A single member of the dive team conducted surveys of sponge biodiversity during eight dives at six locations, at depths ranging from 15 to 30 m. This preliminary assessment documented the presence of 45 species pooled across multiple locations. Rarefaction analysis estimated that only 48 to 84% of species diversity was sampled by this limited effort, clearly indicating a need for additional surveys. An analysis of historical collections from Saba and Saba Bank revealed an additional 36 species, yielding a total of 81 sponge species recorded from this area.

**Conclusions/Significance:**

This observed species composition is similar to that found on widespread Caribbean reefs, indicating that the sponge fauna of Saba Bank is broadly representative of the Caribbean as a whole. A robust population of the giant barrel sponge, *Xestospongia muta*, appeared healthy with none of the signs of disease or bleaching reported from other Caribbean reefs; however, more recent reports of anchor chain damage to these sponges suggests that human activities can have dramatic impacts on these communities. Opportunities to protect this extremely large habitat should be pursued, as Saba Bank may serve as a significant reservoir of sponge species diversity.

## Introduction

Saba Bank (Netherlands Antilles) is one of the three largest atolls on Earth; since this atoll is completely submerged, it provides habitat for an extensive coral reef community [1]–[3]. The total surface area shallower than 200 m is estimated at 2,200 km^2^, with most of this area between 20 and 30 m depth [1], [3]. The vast area of Saba Bank provides critical habitat for economically important fisheries. In 2007, the total value of annual lobster and fish landings was estimated at over $1.6 million [4]. In 1999, this fishery generated nearly 10% of Saba Island's gross domestic product and employed approximately 8% of the island's active workforce [5], [6].

To improve our knowledge of this valuable marine resource, a survey of biodiversity at Saba Bank conducted in January 2006 included a multi-disciplinary team that sampled fishes, mollusks, crustaceans, macroalgae, corals, and sponges. In this volume, Hoetjes and Carpenter [2] provide an overview of the goals of this survey and a review of current threats to this ecosystem. In particular, Saba Bank has become a popular anchorage for large ships travelling to and from oil terminals located on the nearby island of St. Eustatius. Unregulated anchorages can cause dramatic damage by physically destroying benthic organisms and substrates.

Previous expeditions to Saba Bank have documented the presence of diverse coral reef communities [1], [3], with several researchers examining fish, crustacean, and molluscan biodiversity [5], [7]–[10]. However, the biodiversity of other marine invertebrates has not been well reported and there have been no comprehensive assessments of sponge biodiversity at this location. After corals, sponges are the most dominant macroinvertebrates on Caribbean coral reefs [11]–[14], providing ecosystem services such as the stabilization of reef substrates and nutrient cycling between benthic and pelagic components of these environments [14]. Despite these important contributions to ecosystem health and stability, sponges are often overlooked in censuses of marine biodiversity [11].

As sessile, filter-feeding organisms, sponge species and communities are useful biological indicators of water quality [15]–[19]. Reports of sponge diseases have increased in recent years, with anthropogenic disturbances, eutrophication, and organic pollutants commonly cited as proximate causes [20]–[22]. In particular, a major contributor to sponge biomass in the Caribbean, the giant barrel sponge *Xestospongia muta*, has been reported to suffer from diseases at widespread locations [20]. Thus, we examined the health of the conspicuous, large-bodied *X. muta* as an indicator of overall sponge community health.

Although van der Land [1] notes that sponges were collected during the 1972 expedition to Saba Bank, the actual identities of the species present in this collection were not reported. These specimens were deposited at Naturalis, the National Museum of Natural History, Leiden, the Netherlands, as bulk collections and were not identified prior to the current study. During the current study, two additional sets of curated specimens from the island of Saba were located at the Zoological Museum of the University of Amsterdam (ZMA); these records have not been previously published. The goals of our study were to: (1) examine the sponge species composition of the Saba Bank reef communities using both the current survey and historical collections at ZMA and Naturalis; (2) compare the sponge species composition of Saba Bank to other well documented locations in the Caribbean and North Atlantic; and (3) survey the health of a dominant large-bodied sponge, *X. muta*.

## Results

**Figure 1:**
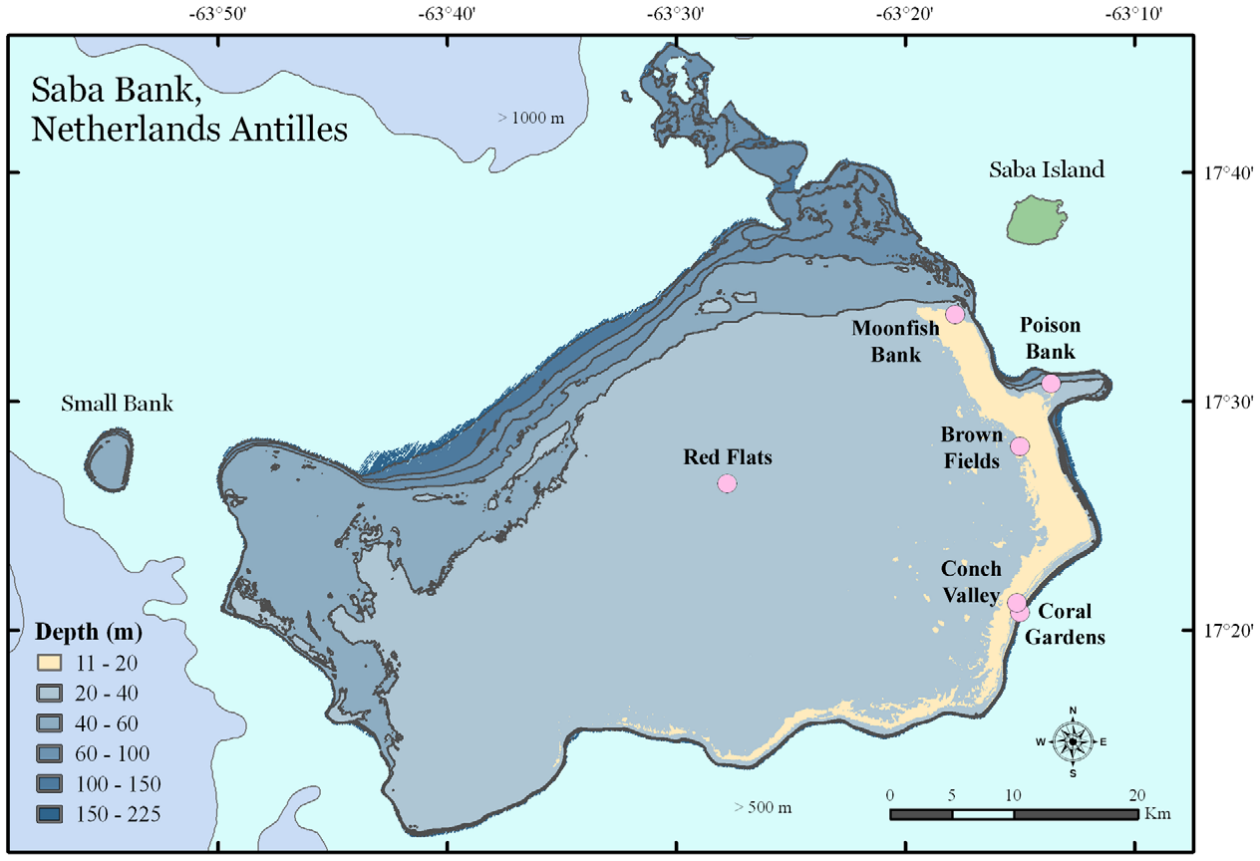
Map of Saba Bank, Netherlands Antilles. Saba Bank is shown relative to its nearest neighbor, Saba Island. A pale band of color representing a “reef crest” at 11 to 20 m depth extends 50 km along the east and southeast perimeter of the Bank. Circles indicate the locations of sponge biodiversity surveys in 2006.

Surveys by a single member of the dive team documented 45 species of sponges at Saba Bank over eight dives at six locations (Table S1; Figure 1). The surveyed sites ranged from 0 to 40% similarity, sharing an average of 15% of the observed sponge species. Species recorded from multiple locations included: *Agelas clathrodes*, *Agelas conifera*, *Agelas dispar*, *Aiolochroia crassa*, *Amphimedon compressa*, *Aplysina cauliformis*, *Halichondria melanodocia*, *Monanchora arbuscula*, *Mycale laevis*, *Neopetrosia carbonaria*, *N. subtriangularis*, *Plakortis halichondrioides*, *Spheciospongia vesparium*, *Verongula rigida*, and *Xestospongia muta*. During these surveys, 36 individuals of *X. muta* were encountered; no individuals showed signs of disease or damage.

**Figure 2:**
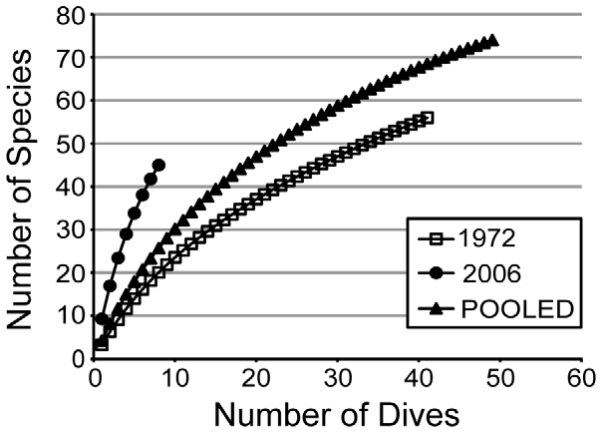
Species accumulation curves for sponges observed on Saba Bank by expeditions in 1972 (open squares), 2006 (filled circles), and the two expeditions pooled together (filled triangles). Each curve represents Mao Tau (S_obs_) values of species richness plotted against the number of dives on which sponges were observed. Since these species accumulation curves do not approach clear asymptotes, more sponge species are likely to be discovered on Saba Bank.

Rarefaction analyses suggested that the 45 species observed during the 2006 surveys described only 69% (95% confidence interval: 48 to 84%) of the Chao2 estimate of species diversity (65 species; 95% confidence interval: 54 to 93; Figure 2). Our identification of specimens in the van der Land [1] collection from the 1972 expedition to Saba Bank revealed 56 species, with 29 of these not represented in the 2006 surveys (Table S1). Only 18 species found in 2006 were not present in the 1972 collections, while 27 species were reported by both expeditions. The Vermeulen 1986 collection from Saba contained an additional seven unique species records, for a total of 81 species documented from this region (Table S1).

Rarefaction analyses suggested that the 41 dives conducted by the 1972 expedition observed only 48% (95% confidence interval: 26 to 71%) of the Chao2 estimate of species diversity (116 species; 95% confidence interval: 79 to 213; Figure 2). When data from 1972 and 2006 were pooled, the combined surveys are estimated to describe 75% (95% confidence interval: 56 to 88%) of the Chao2 estimate of species diversity (97 species; 95% confidence interval: 83 to 131; Figure 2). None of the three rarefaction curves approaches an asymptote (Figure 2), suggesting that many more sponge species are likely to be reported from Saba Bank.

**Figure 3:**
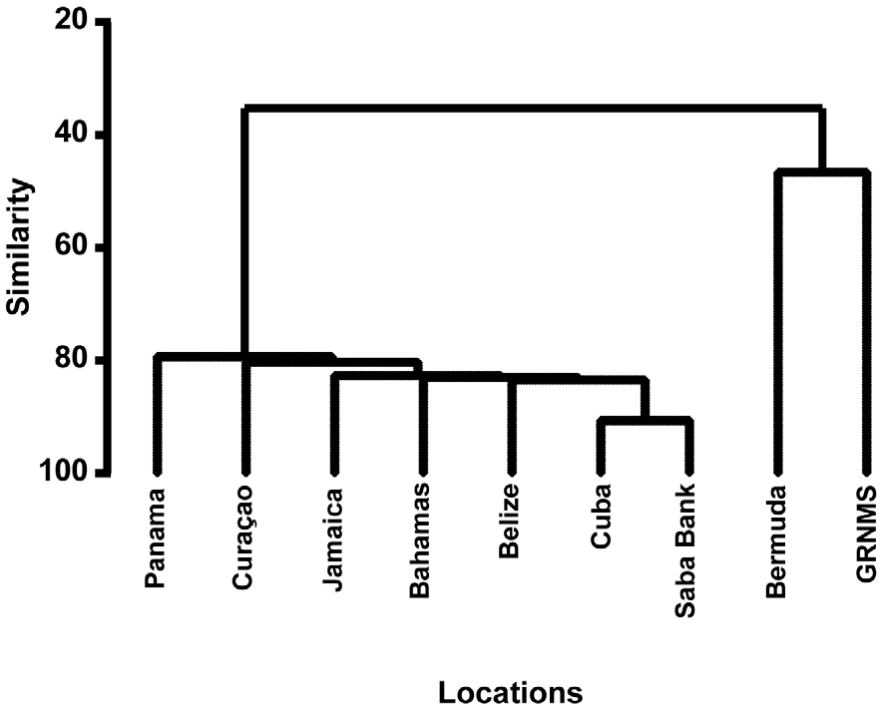
Hierarchical clustering dendrogram of similarity in sponge species composition between Saba Bank and eight other locations. The Caribbean locations were not significantly different from each other, but were significantly different from the North Atlantic locations of Bermuda and GRNMS. Panama  =  Bocas del Toro region of Panama; GRNMS  =  Gray's Reef National Marine Sanctuary, Georgia, USA.

When comparing species composition among Caribbean locations, similarity ranged from 75% to 91%, with no statistically significant variation among these broadly distributed areas (Pi = 0.488, P = 0.699, Figure 2). The Caribbean locations displayed significant differences in species composition (30% to 43% similarity) when compared to the North Atlantic communities of Gray's Reef and Bermuda (Pi = 17.565, P<0.001, Figure 3), which showed 47% similarity to each other.

## Discussion

Our combination of SCUBA-based surveys and analyses of historical collections documented a total of 81 sponge species from Saba and Saba Bank. This number most likely underestimates the actual sponge species diversity present. In comparison to other recent surveys of sponge biodiversity in the Caribbean, Saba Bank shows lower diversity than Rützler et al.'s [23] survey of mangrove cays in Belize (182 species) and lower diversity than Diaz's [24] survey of the Bocas del Toro region of Panama (120 species). However, both of these studies used much greater sampling effort than the current survey. In Belize, six individual collectors snorkeled seven sites over a two-week time period [23], while in Panama, Diaz worked as a single collector for approximately one hour at each of 14 sites [24]. By contrast, Alcolado's [25] comprehensive catalog of the sponges of Cuba lists 280 species in a compilation of multiple surveys over the past 140 years conducted by at least 12 different investigators. Since the 2006 surveys at Saba Bank were performed by a single observer and were time-limited, additional survey efforts will most likely reveal greater sponge biodiversity than we have currently documented. In addition, the use of quantitative survey techniques (e.g., measuring relative abundance at each site) will allow future researchers to quantify variation in sponge communities across different locations on Saba Bank and to relate this variability to other environmental parameters.

Sponge species composition at Saba Bank is broadly representative of the Caribbean sponge fauna as a whole. When comparing Saba Bank to six other well-documented locations throughout the Caribbean, there are no statistically significant differences in species composition. Given that these locations showed 75–91% similarity, these communities can be viewed as a sharing a core group of widely distributed species. However, it is important to note that no one location held all of the same species. Since each of the six other Caribbean locations also contains unique and potentially endemic sponge species, further exploration of Saba Bank might also reveal additional species that are new to science.

**Figure 4:**
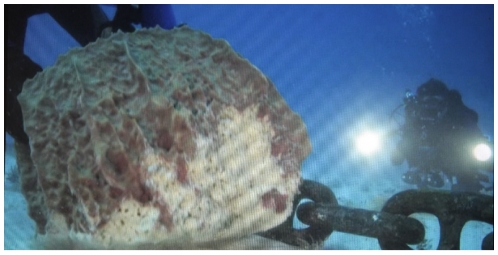
Anchor chain damaging a giant barrel sponge, *Xestospongia muta*. Large anchor chains of oil tankers and shipping vessels can rapidly damage coral reef habitats. Note the presence of divers for scale. Still frame courtesy of Robin Waite, Yap Films Inc.

The sponge fauna of Saba Bank appeared particularly healthy in 2006, with no signs of disease or damage in the giant barrel sponge *X. muta*. Because of its distance from large landmasses, Saba Bank may receive fewer impacts from typical, waterborne anthropogenic environmental stressors compared to other Caribbean reef ecosystems [15]. However, recently we have received multiple reports of anchor chain damage to individuals of *X. muta* (Figure 4). As these chains drag across benthic habitats, they can dislodge sponges from the substrate, often fragmenting and crushing them. These findings are distressing since large *X. muta* individuals, often termed the “redwoods of the reef,” can be hundreds of years old [26]. Restoration efforts that reattach broken *X. muta* to the substrate can be successful [27], but these are costly in terms of personnel effort. Moreover, the many smaller-bodied sponge species that are damaged or destroyed by the movement of anchor chains cannot be restored using such techniques.

Maintaining a healthy, diverse community of sponges at Saba Bank should be a goal of any future management plans for this region. These communities provide critical ecosystem services, including habitat and nutrition for a variety of fish and invertebrate species [11]–[14]. Further, sponges can also provide early indicators of water quality and ecosystem health [15], [18]. As stressed by Meesters et al. [3], Saba Bank may represent an important source of larval dispersal to reefs throughout the Caribbean. The large area and wide variety of Saba Bank's reefs [1], suggest that these communities provide an important reservoir of biodiversity for the Caribbean as a whole.

## Materials and Methods

### Ethics Statement

All Saba Bank projects have collecting permits through CITES (where necessary) and the Saba Conservation Foundation (where CITES is not required).

### Surveys

Benthic communities at depths ranging from 15 m to 30 m were surveyed during eight dives at six sites along the eastern portion of the Saba Bank in January 2006 (Figure 1). A regional map and additional descriptions of each site are provided in this volume by Hoetjes and Carpenter [2]. Sites to be assessed were chosen based on coarse resolution bathymetry, proximity to Saba Island, Van der Land's description [1], and qualitative information from reconnaissance dives. Given the large area of Saba Bank, the sites were intended to cover a range of localities with a focus on known or hypothesized reef areas. Reef areas were assigned top priority for sampling due to the likely presence of corals and their high susceptibility to physical damage from maritime activity. Known reef areas occur on the east, southeast, and southern edges of the bank. The deeper western side of Bank could potentially have patch reefs where ridges less than 30 m deep occur. Weather was an important factor during the survey period of 4–15 January 2006, as mean wave heights of 3 m–4.5 m were recorded on several days.

While other dive team members focused on scleractinian corals, soft corals, fishes, or macroalgae, a single member of the dive team documented the presence of sponge species, with 20 to 45 minutes of survey time at each location. Presence of each species was recorded on underwater paper and by digital photography. It is important to note that the lack of a “presence” observation does not necessarily indicate that a species is “absent” at a particular location, as these surveys were not quantitative. Specimens representing a subset of the observed species were collected for taxonomic verification using microscopic morphological characters [28]. Encountered individuals of the giant barrel sponge, *Xestospongia muta*, were examined for evidence of damage or disease.

### Historical Collections

In 1972, a survey of Saba Bank was conducted by the hydrographic vessel H.NL.M.S. LUYMES of the Royal Dutch Navy as part of the international Co-operative Investigations of the Caribbean and Adjacent Regions (CICAR) Program [1]. The sponges collected by divers of the Royal Dutch Navy and Van Veen grabs during this survey were not fully documented, but were retained at Naturalis, the National Museum of Natural History, Leiden the Netherlands, as a bulk collection of approximately 220 specimens. This collection was located and sorted by species using morphological characters. Additional sponges from the 1972 survey and from the island of Saba were located at the Zoological Museum of Amsterdam (ZMA). In 1963, two specimens were deposited in the museum by P. Wagenaar Hummelinck, while in 1986, 35 specimens were deposited by J.J. Vermeulen. The species represented in these historical collections were added to those encountered during the 2006 survey to generate a more complete list of species composition (Table S1).

### Data Analyses

The number of sponge species recorded among dives was assessed using rarefaction analyses, with the Chao2 estimator [29] serving as an estimate of total species diversity. Rarefaction curves and the Chao2 expected species richness were calculated using EstimateS software [30]. Rarefaction curves were plotted using the cumulative number of species observed (Mao Tau, [30]) on each dive for the 1972 data alone, the 2006 data alone, and the pooled 1972 and 2006 data. An estimate of the percentage of total species richness that was documented was obtained by dividing the observed number of species by the Chao2 estimated species richness.

The species composition of Demospongiae at Saba Bank was compared to other well-documented locations in the Caribbean, including the Bahamas [31], Belize [23], Cuba [25], Curaçao [32], Jamaica [33]–[35], and Panama [24]. We also included two North Atlantic locations in these comparisons, Bermuda [36] and Gray's Reef National Marine Sanctuary (GRNMS) [37]. A presence/absence matrix was created to compare the sponge species documented at Saba Bank with those from the other eight locations. These data were analyzed using the PRIMER software package (Plymouth Routines in Multivariate Ecological Research, version 6) [38]. Bray-Curtis similarity values were calculated and used to generate dendrograms displaying hierarchical clusters of similar sites by the group averages method. The SIMPROF procedure was used to test the null hypotheses of (1) no differences among all sites and (2) no differences among Caribbean sites, exclusive of the two North Atlantic sites (Bermuda and GRNMS). Since two comparisons were made with these data, the level of significance was set to 0.025; 1000 permutations of the data set were used to assess significance.

## Supporting Information

Table S1 Sponge species documented at the island of Saba and the Saba Bank atoll during expeditions in 1972 [1] (deposited at Naturalis), 1986 (collected by J.J. Vermeulen; deposited at ZMA), and 2006 (current study).
(0.17 MB DOC)
