# Supplementary material for: Preliminary Assessment of Sponge Biodiversity on Saba Bank, Netherlands Antilles
Source: PLoS One. 2010 May 21;5(5):e9622. doi: 10.1371/journal.pone.0009622 (PMC2873950; doi:10.1371/journal.pone.0009622)
Supplement: Table S1 — Sponge species documented at the island of Saba and the Saba Bank atoll during expeditions in 1972 [1] (deposited at Naturalis), 1986 (collected by J.J. Vermeulen; deposited at ZMA), and 2006 (current study). (0.17 MB DOC) [file pone.0009622.s001.doc]

| Class | Order | Family | Genus-species-Author-Year | Year of Expedition | | |
| --- | --- | --- | --- | --- | --- | --- |
| **Calcarea** | |  |  | 1972 | 1986 | 2006 |
|  | Clathrinida | Leucettidae | *Leucetta floridana* Haeckel, 1872 | 46, 124, 136 |  |  |
| **Demospongiae** | |  |  |  |  |  |
|  | Agelasida | Agelasidae | *Agelas clathrodes* (Schmidt, 1870) |  | FB | MB, PB |
|  | Agelasida | Agelasidae | *Agelas conifera* (Schmidt, 1870) | 124, 126, 144, 153 |  | CG, CV, MB, PB |
|  | Agelasida | Agelasidae | *Agelas dispar* Duchassaing & Michelotti, 1864 | 45, 46, 67, 74, 115, 124, 134, 144 |  | MB, PB |
|  | Agelasida | Agelasidae | *Agelas sventres* Lehnert & van Soest, 1996 |  | FB |  |
|  | Astrophorida | Ancorinidae | *Ancorina* sp. | 15 |  |  |
|  | Astrophorida | Ancorinidae | *Stelletta stenospiculata* Uliczka, 1929 | 132 |  |  |
|  | Astrophorida | Geodiidae | *Erylus formosus* Sollas, 1886 |  |  | BF |
|  | Astrophorida | Geodiidae | *Geodia gibberosa* Lamarck, 1815 |  | FB |  |
|  | Dendroceratida | Dictyodendrillidae | *Igernella notabilis* (Duchassaing & Michelotti, 1864) | 46 |  |  |
|  | Dictyoceratida | Dysideidae | *Dysidea etheria* de Laubenfels, 1936 | 136 |  | MB |
|  | Dictyoceratida | Dysideidae | *Dysidea fragilis* (Montagu, 1818) |  |  | RF |
|  | Dictyoceratida | Irciniidae | *Ircinia campana* (Lamarck, 1814) | 55, 74 | FB | CV |
|  | Dictyoceratida | Irciniidae | *Ircinia felix* (Duchassaing & Michelotti, 1864) | 29, 46, 55, 100, 122, 124, 153 | FB | MB |
|  | Dictyoceratida | Irciniidae | *Ircinia strobilina* (Lamarck, 1816) | 212 | FB | MB |
|  | Dictyoceratida | Spongiidae | *Spongia (Spongia) pertusa* Hyatt, 1877 | 32 |  | RF |
|  | Dictyoceratida | Thorectidae | *Hyrtios proteus* Duchassaing & Michelotti, 1864 |  | FB | MB |
|  | Dictyoceratida | Thorectidae | *Hyrtios violaceus* (Duchassaing & Michelotti, 1864) | 55, 124, 136 |  |  |
|  | Hadromerida | Clionaidae | *Cervicornia cuspidifera* (Lamarck, 1815) | 55, 136 |  |  |
|  | Hadromerida | Clionaidae | *Cliona varians* (Duchassaing & Michelotti, 1864) | 32, 42 |  | MB |
|  | Hadromerida | Clionaidae | *Spheciospongia vesparium* (Lamarck, 1815) | 53, 59 |  | MB, RF |
|  | Hadromerida | Clionaidae | *Spheciospongia* sp. |  | FB |  |
|  | Hadromerida | Suberitidae | *Prosuberites laughlini* (Diaz, Alvarez & van Soest, 1987) | 46, 126 |  |  |
|  | Hadromerida | Suberitidae | *Terpios fugax* Duchassaing & Michelotti, 1864 |  |  | MB |
|  | Hadromerida | Tethyidae | *Tectitethya crypta* (de Laubenfels, 1949) | 136 |  | RF |
|  | Hadromerida | Timeidae | *Timea unistellata* (Topsent, 1892) |  |  | MB |
|  | Halichondrida | Axinellidae | *Dragmacidon explicatum* (Wiedenmayer, 1977) | 55, 100,126 |  |  |
|  | Halichondrida | Axinellidae | *Dragmacidon reticulatum* (Ridley & Dendy, 1886) |  | FB |  |
|  | Halichondrida | Axinellidae | *Phakellia folium* Schmidt, 1970 | 101 |  |  |
|  | Halichondrida | Axinellidae | *Ptilocaulis walpersi* (Duchassaing & Michelotti, 1864) | 40, 55, 62, 121, 126 | FB |  |
|  | Halichondrida | Halichondriidae | *Halichondria (Halichondria) lutea* Alcolado, 1984 | 126 |  |  |
|  | Halichondrida | Halichondriidae | *Halichondria (Halichondria) magniconulosa* Hechtel, 1965 |  |  | CG |
|  | Halichondrida | Halichondriidae | *Halichondria (Halichondria) melanodocia* de Laubenfels, 1936 |  |  | CG |
|  | Halichondrida | Halichondriidae | *Topsentia bahamensis* Diaz, van Soest & Pomponi, 1993 | 101 |  |  |
|  | Halichondrida | Heteroxyidae | *Myrmekioderma gyroderma* (Alcolado, 1984) |  |  | RF |
|  | Halichondrida | Heteroxyidae | *Myrmekioderma rea* (de Laubenfels, 1934) | 126 |  |  |
|  | Haplosclerida | Callyspongiidae | *Callyspongia (Callyspongia) fallax* Duchassaing & Michelotti, 1864 | 55 | FB |  |
|  | Haplosclerida | Callyspongiidae | *Callyspongia (Cladochalina) armigera* (Duchassaing & Michelotti, 1864) | 124 | FB |  |
|  | Haplosclerida | Callyspongiidae | *Callyspongia (Cladochalina) plicifera* (Lamarck, 1814) | 144 |  | CG |
|  | Haplosclerida | Callyspongiidae | *Callyspongia (Cladochalina) vaginalis* (Lamarck, 1814) | 84, 100, 102, 114, 121, 153 | FB | MB |
|  | Haplosclerida | Niphatidae | *Amphimedon caribica* (Pulitzer-Finali, 1986) | 67, 114, 115, 126, 146 |  |  |
|  | Haplosclerida | Niphatidae | *Amphimedon compressa* Duchassaing & Michelotti, 1864 | 46, 62, 69, 111, 121, 124, 153 |  | BF, CV, MB |
|  | Haplosclerida | Niphatidae | *Amphimedon erina* (de Laubenfels, 1936) |  |  | MB |
|  | Haplosclerida | Niphatidae | *Amphimedon viridis* Duchassaing & Michelotti, 1864 | 78 | SB |  |
|  | Haplosclerida | Niphatidae | *Niphates amorpha* Wiedenmayer, 1977 | 122 |  |  |
|  | Haplosclerida | Niphatidae | *Niphates digitalis* (Lamarck, 1814) | 46, 55, 100, 111, 120, 121, 124, 144, 146, 153 | FB | MB |
|  | Haplosclerida | Niphatidae | *Niphates erecta* Duchassaing & Michelotti, 1864 | 69, 126 |  | MB |
|  | Haplosclerida | Petrosiidae | *Neopetrosia carbonaria* (Lamarck, 1814) |  | FB | MB, PB |
|  | Haplosclerida | Petrosiidae | *Neopetrosia subtriangularis* (Duchassaing, 1850) | 115, 124 |  | BF, CV, MB |
|  | Haplosclerida | Petrosiidae | *Petrosia (Petrosia) weinbergi* van Soest, 1980 | 101 |  |  |
|  | Haplosclerida | Petrosiidae | *Xestospongia muta* (Schmidt, 1870) | 89, 121, 136 |  | BF, MB |
|  | Haplosclerida | Phloeodictyidae | *Aka xamaycaensis* (Pulitzer-Finali, 1986) | 115 |  |  |
|  | Haplosclerida | Phloeodictyidae | *Calyx podatypa* (de Laubenfels, 1934) | 115 | FB |  |
|  | Haplosclerida | Phloeodictyidae | *Oceanapia peltata* (Schmidt, 1870) | 132 |  |  |
|  | Homosclerophorida | Plakinidae | *Plakortis angulospiculatus* (Carter, 1882) |  | FB |  |
|  | Homosclerophorida | Plakinidae | *Plakortis halichondrioides* (Wilson, 1902) |  |  | CG, PB, MB |
|  | Lithistida | Desmanthidae | *Petromica (Chaladesma) ciocalyptoides* (van Soest & Zea, 1986) | 136 |  |  |
|  | Poecilosclerida | Crambeidae | *Monanchora arbuscula* (Duchassaing & Michelotti, 1864) | 55 |  | BF, MB |
|  | Poecilosclerida | Desmacididae | *Desmapsamma anchorata* (Carter, 1882) | 131, 132 | FB |  |
|  | Poecilosclerida | Desmacellidae | *Neofibularia nolitangere* (Duchassaing & Michelotti, 1864) | 153 | FB | MB |
|  | Poecilosclerida | Hymedesmiidae | *Phorbas amaranthus* Duchassaing & Michelotti, 1864 |  |  | RF |
|  | Poecilosclerida | Iotrochotidae | *Iotrochota birotulata* (Higgin, 1876) | 121 | FB | MB |
|  | Poecilosclerida | Microcionidae | *Clathria (Thalysias) juniperina* (Lamarck, 1814) | 115 |  | RF |
|  | Poecilosclerida | Microcionidae | *Pandaros acanthifolium* Duchassaing & Michelotti, 1864 | 69 |  |  |
|  | Poecilosclerida | Mycalidae | *Mycale (Arenochalina) laxissima* (Duchassaing & Michelotti, 1864) |  |  | MB |
|  | Poecilosclerida | Mycalidae | *Mycale (Mycale) laevis* (Carter, 1882) |  |  | CG, MB |
|  | Poecilosclerida | Raspailiidae | *Ectyoplasia ferox* (Duchassaing & Michelotti, 1864) | 115 | FB | BF |
|  | Poecilosclerida | Tedaniidae | *Tedania (Tedania) ignis* (Duchassaing & Michelotti, 1864) | 52, 53, 126,136 | FB |  |
|  | Poecilosclerida | Tedaniidae | *Tedania (Tedania) klausi* Wulff, 2006 | 131 |  |  |
|  | Spirophorida | Tetillidae | *Cinachyrella alloclada* (Uliczka, 1929) |  |  | MB |
|  | Spirophorida | Tetillidae | *Cinachyrella arenosa* (van Soest & Stentoft, 1988) | 43, 126, 136 |  |  |
|  | Spirophorida | Tetillidae | *Cinachyrella kuekenthali* (Uliczka, 1929) | 32, 46, 121, 153 |  | MB |
|  | Verongida | Aplysinidae | *Aiolochroia crassa* (Hyatt, 1875) | 46, 85, 144 | FB | BF, MB |
|  | Verongida | Aplysinidae | *Aplysina archeri* (Higgin*, 1875)* | 153 |  |  |
|  | Verongida | Aplysinidae | *Aplysina cauliformis* (Carter, 1882) | 46, 59, 62, 67, 84, 98, 100, 102, 111, 115, 122 | FB | CV, MB |
|  | Verongida | Aplysinidae | *Aplysina fistularis* (Pallas, 1766) | 72 |  | MB |
|  | Verongida | Aplysinidae | *Aplysina fulva* (Pallas, 1766) | 53, 55, 144 | FB | MB |
|  | Verongida | Aplysinidae | *Aplysina insularis* (Duchassaing & Michelotti, 1864) |  | FB |  |
|  | Verongida | Aplysinidae | *Aplysina lacunosa* (Pallas, 1766) |  | FB | PB |
|  | Verongida | Aplysinidae | *Verongula gigantea* (Hyatt, 1875) |  | FB |  |
|  | Verongida | Aplysinidae | *Verongula rigida* (Esper, 1794) |  |  | BF, MB |

Two additional specimens were collected by P. Wagenaar Hummelinck in 1963 at Fort Bay and deposited at ZMA: *Ircinia felix* and *Niphates amorpha*; both species were also reported by subsequent surveys. For each expedition, the locality of collection is indicated; for the 1972 expedition, localities are described by Van der Land [1].

BF = Brown Fields (1 dive); 17°28.03 N, 63°14.94 W.

CG = Coral Gardens (1 dive) 17°20.76 N, 63°15.04 W.

CV = Conch Valley (1 dive) 17°21.18 N, 63°15.12 W.

FB = Fort Bay, Saba Island; 800 m from landing stage; collected using snorkel; approximately 17°36.97 N, 63°15.11 W.

MB = Moonfish Bank (3 dives); 17°33.77 N, 63°17.90 W.

PB = Poison Bank (1 dive); 17°30.76 N, 63°13.69 W.

RF = Red Flats (1 dive); 17°26.36 N, 63°27.77 W.

SB = Saba Bank, with no other specific locality recorded.
